# Waveform engineering analysis of photoacoustic radar chirp parameters for spatial resolution and SNR optimization

**DOI:** 10.1016/j.pacs.2019.04.003

**Published:** 2019-05-02

**Authors:** Zuwen Sun, Natalie Baddour, Andreas Mandelis

**Affiliations:** a Department of Mechanical Engineering, University of Ottawa, 161 Louis Pasteur, K1N 6N5, Ottawa, Canada; b Center for Advanced Diffusion-Wave and Photoacoustic Technologies (CADIPT), Department of Mechanical and Industrial Engineering, University of Toronto, 5 King’s College Road, M5S 3G8, Toronto, Canada; c Institute of Biomaterials and Biomedical Engineering, University of Toronto, Canada

**Keywords:** Frequency-domain photoacoustics, Pulse compression, Autocorrelation, Matched-filter

## Abstract

Recent developments in photoacoustics have witnessed the implementation of a radar matched-filtering methodology into the continuous wave photoacoustic modality. The main merit of using matched filtering in continuous photoacoustics is the improvement in signal to noise ratio (SNR), but the correlation process may result in a loss of resolution. It is possible to enhance both SNR and resolution by matched-filtering and pulse compression with a frequency chirp. However, the theory behind the effect of the chirp parameters on both SNR and resolution is still not clear. In this paper, the one-dimensional theory of the photoacoustic radar with a pulse compressed linear frequency modulated sinusoidal laser chirp is developed. The effect of the chirp parameters on the corresponding photoacoustic signal is investigated, and guidelines for choosing the chirp parameters for resolution and SNR optimization are given based on theory and simulations. The results show that by judiciously manipulating the center frequency, bandwidth, and duration, the resolution and SNR can be easily enhanced.

## 1 Introduction

Frequency-domain photoacoustic (FD-PA) imaging for biomedical applications has attracted interest over the past decade, with important applications still under development [[1], [2], [3], [4]]. This imaging modality works through irradiation of an absorbing material (absorber, or chromophore) by a laser source. The energy absorbed produces a small temperature rise, which induces excess pressure inside the sample through thermoelastic expansion. This pressure acts as an acoustic source and generates further acoustic waves, which can be detected by ultrasound transducers positioned outside the sample. Since there is a large difference in optical absorption between blood and surrounding tissue, the ultrasound wave induced by laser irradiation carries information about the optical absorption properties of blood bearing tissue. This approach is thus suitable for the imaging of the micro-vascular system or for tissue characterization.

The most common excitation source for photoacoustics has been pulsed electromagnetic waves, for example in the work by Kruger [5,6] and Wang [[7], [8], [9]]. The key advantage of using a short pulse to irradiate the tissue is that the distribution of heat sources can be directly ascertained from the shape of the photoacoustic response signal [10]. However, there are still challenges to implement pulse photoacoustics. For example, the pulsed laser modality is limited by incident energy levels that must meet safety standards for in-vivo tissue imaging [11]. The short nanosecond incident pulse will generate a wide-band PA signal which requires a wide band transducer for detection. Moreover, expensive and bulky Q-switched laser source, wide-bandwidth noise, as well as the presence of often large signal baselines in pulsed photoacoustics are also pushing researchers’ interest to continuous wave photoacoustics [11,12].

An alternative excitation modality that has also been proposed is FD-PA, where the acoustic wave is generated by periodic modulation of a laser [[12], [13], [14], [15]]. More recently, the idea of implementing a pulse compression approach via matched filtering was introduced and investigated [12,[16], [17], [18], [19], [20], [21], [22]], often referred to as the Photoacoustic Radar (PAR). The matched filter approach enables detection of a known signal immersed in Gaussian white noise, therefore a long duration coded waveform with moderate power could potentially replace short high-power pulses.

The major chirp modulated PAR advantages over conventional pulsed laser PA imaging modalities are: 1) substantially higher image acquisition frame rates (kHz) than Q-switched pulsed lasers, enabling practical real-time clinical imaging, 2) small diode laser source footprint with the potential for portable multi-wavelength imaging applications, 3) two images (amplitude and phase-based) instead of one at each probed subsurface depth for higher diagnostic power, 4) depth selectivity via cross-correlation delay-time fixing (quick tomographic slice localization and operator-controlled fixed-depth image formation), 5) comparable imaging axial resolution and SNR, 6) potential for real-time, baseline absorption and signal distortion eliminating, differential wavelength imaging. These features of PAR imaging using inexpensive laser diodes and standard ultrasound transducers offer the exciting possibility and unique opportunity of developing novel, portable commercial clinical and preclinical co-registered ultrasonic-photoacoustic (US-PA) imaging systems, with sub-mm axial resolution and optical-level contrast with the aforementioned advantages over pulsed laser PA imagers.

Signal to noise ratio (SNR), contrast, resolution, and depth sensitivity are several aspects that need to be evaluated in order to assess the performance of an imaging system. Different approaches have been investigated to improve the performance of FD-PA, such as using a contrast agent to improve the contrast of the image [23], using coherent or incoherent averaging signal processing methods to increase the SNR [12], and optimizing chirp parameters to improve SNR [20]. However, the detailed theory behind optimizing the chirp parameters for PAR is still not fully developed.

Recent research [24] showed that a chirp excitation PA system may have lower SNR than the pulsed PA system. However, the effect of the chirp parameters are still not clear. Lashkari and Mandelis have investigated the effect of chirp parameters on the SNR of PAR [20]. Their experimental results showed that the chirp sweep range is one of the key parameters that affect SNR. They demonstrated that the optimal chirp sweep range tends to be in the low MHz range. For a frequency transducer with 3.5 MHz center frequency, they found that the optimal chirp bandwidth was 0.5–3 MHz, chosen from amongst three different sweeping ranges (0.5–3 MHz, 1–3 MHz, 0.5–5 MHz). For a lower frequency transducer (0.5 MHz center frequency), the optimal chirp bandwidth was demonstrated to be 200–850 kHz. Keeping the lower cutoff frequency of the chirp constant and increasing the upper limit did not increase SNR significantly. Thus, they demonstrated that there is a certain optimal bandwidth that can produce the best SNR. However, the derived mathematical expressions do not fully explain this phenomenon as only the effect of the chirp duration was considered in the theory.

In PAR, the profile of the cross-correlated signal (sometimes called A-scan profile) is also an important way to obtain information about an absorber. In most studies, the cross-correlated signal has only one recognizable peak that reveals the front edge of the absorber (the absorber surface which is near the transducer) [17,19,20]. The theoretical and experimental results in [12] have two peaks representing the front and rear edge of the absorber. However, the profiles of the absorbers were not fully obtained.

Although the photoacoustic radar promises to be an important development towards overcoming the limitations of the short-pulse approach, a generalized theory still remains to be developed. In this paper, such a one-dimensional theory is developed, and the effects of the chirp parameters on SNR and resolution are investigated. Additionally, the cross-correlated signal profiles are discussed in relation to the chirp parameters.

## 2 Formulation of the problem

### 2.1 Physical model and Fourier shell theorem

The governing equation for a PA wave is given by

(1) $$\left[\nabla^{2}-\frac{1}{c_{s}^{2}}\frac{\partial^{2}}{\partial t^{2}}\right]p\left(\vec{r},t\right)=-\frac{p_{0}}{c_{s}^{2}}A\left(\vec{r}\right)\frac{\partial I(t)}{\partial t}$$

where $p_{0}=\frac{\beta c_{s}^{2}}{C_{p}}\mu_{a}F$ and β is the thermal expansion coefficient, $c_{s}$ is the speed of sound, $C_{p}$ is the specific heat, $\mu_{a}$ is the optical absorption coefficient of the chromophore absorber that has been heated by an optical pulse with fluence *F*. $p(\vec{r},t)$ is the pressure of the acoustic wave, a function of space and time. $A\left(\vec{r}\right)$ is a function of space that describes the geometry of the absorber and $I\left(t\right)$ is a function that describes the time dependence of the incident optical pulse. Diebold [25] gives a concise explanation of the governing equation for the pressure that results from launching a photoacoustic wave.

In this paper, our focus is on the spectral analysis of the design of the input waveform $I\left(t\right)$, therefore to simplify geometrical effects, we consider a one-dimensional Cartesian space, where position is a function of *z* only, so $\vec{r}=z$ Taking the temporal Fourier transform (denoted with a tilde) and then a spatial Fourier transform (indicated as an overhat) in the spatial variable *z* transforms *z* to the spatial frequency variable $\omega_{z}$, and Eq. (1) becomes

(2) $$\hat{\tilde{p}}\left(\omega_{z},\omega \right)=\frac{ikp_{0}}{c_{s}}\tilde{I}\left(\omega \right)\frac{\hat{A}\left(\omega_{z}\right)}{\omega_{z}^{2}-k^{2}}$$

where $k=\omega /c_{s}$ is the angular wavenumber. It has previously been shown [26] via inverse spatial Fourier transformation of Eq. (2) that the pressure response in the temporal frequency domain to a source $I\left(t\right)$ with Fourier transform $\tilde{I}\left(\omega \right)$, and inhomogeneity $A\left(z\right)$ with spatial Fourier transform $\hat{A}\left(\omega_{z}\right)$ is given by

(3) $$\tilde{p}\left(z,\omega \right)=\frac{p_{0}}{2c_{s}}\tilde{I}\left(\omega \right)\left\{\begin{array}{c} \hat{A}\left(-k\right)e^{-ikz}\;\;\;\;\;z>0\\ \hat{A}\left(k\right)e^{ikz}\;\;\;\;\;\;\;\;\;z<0 \end{array}\right)$$

Here, z>0 is to be interpreted as measurements in a transmission mode and z<0 as measurements made in reflection. Eq. (3) is the 1D statement of the Fourier shell theorem for photoacoustics. For Eq. (3), it is assumed that $\hat{A}\left(\omega_{z}\right)$ has no poles. If it does, a simple partial fraction decomposition of $\hat{A}\left(\omega_{z}\right)$ can be used in Eq. (2) prior to the application of the spatial inverse Fourier transform. Further analysis has shown that even in the case that $\hat{A}\left(\omega_{z}\right)$ has a pole, Eq. (3) is still valid outside the region of inhomogeneity (where a detector would be placed).

### 2.2 Transfer function and impulse response

**Fig. 1:**
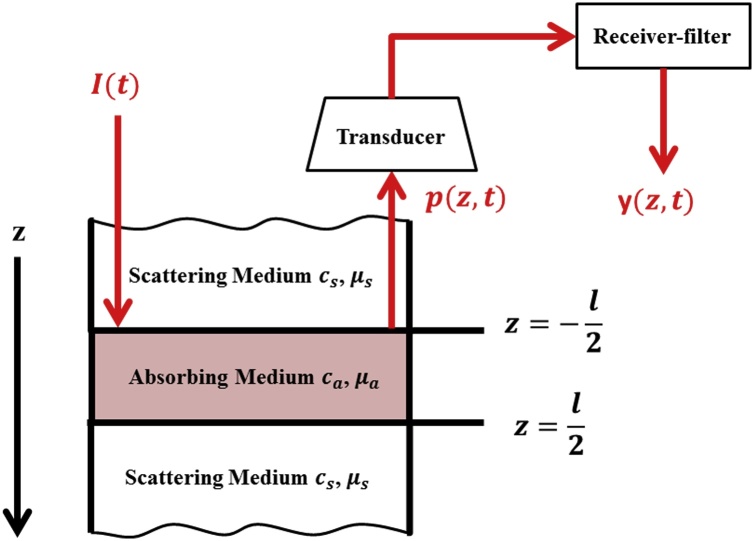
Photoacoustic system model.

A common method of studying linear processes is to view them as linear systems and to study the input/output relationships. The system impulse response or equivalently the transfer function/frequency response are then convenient tools for characterizing input/output relationships. To apply linear systems analysis to photoacoustic problems, input and output quantities need to be defined. The photoacoustic system model is shown in Fig. 1. This represents an absorbing inclusion surrounded by scattering turbid tissue. We define the input quantity to be the input optical pulse given by $I\left(t\right)$ and the output as the pressure response time function measured at some fixed point, *z*, in space, given by $p\left(z,t\right)$. The input/output relationship can then be interpreted as being given by Eq. (3), which may be written in input/output (transfer function) form in the frequency domain as the product

(4) $$\tilde{p}\left(z,\omega \right)=\tilde{G}\left(z,\omega \right)\tilde{I}\left(\omega \right)$$

where the transfer function is given by

(5) $$\tilde{G}\left(z,\omega \right)=\frac{p_{0}}{2c_{s}}\left\{\begin{array}{c} \hat{A}\left(-k\right)e^{-ikz}\;\;\;\;\;z>0\\ \hat{A}\left(k\right)e^{ikz}\;\;\;\;\;\;\;\;\;z<0 \end{array}\right)$$

Eq.s (4) and (5) clearly show that the transfer function is, unsurprisingly, completely controlled by the shape of the absorber, $\hat{A}\left(k\right)$.

The system impulse response can be computed via inverse temporal Fourier transformation of the transfer function $\tilde{G}\left(z,\omega \right)$

(6) $$g\left(z,t\right)=\frac{p_{0}}{2c_{s}}\left\{\begin{array}{c} A\left(z-c_{s}t\right)\;\;\;\;\;\;\;\;z>0\\ A\left(z+c_{s}t\right)\;\;\;\;\;\;\;\;z<0 \end{array}\right)$$

Eq. (6) demonstrates that the temporal impulse response has exactly the same functional form as the spatial shape of the inhomogeneity, although at a fixed measurement location *z* it is a function of time, whereas the shape of the absorber is a function of space. The two responses are related through the speed of sound converting factor. This confirms results phrased in terms of retarded time by Diebold [25].

For an arbitrary input waveform $I\left(t\right)$, Eq. (4) can also be computed in the time domain as a convolution of the input $I\left(t\right)$ with the system impulse response $g\left(z,t\right)$

(7) $$p\left(z,t\right)=\int_{-\infty }^{\infty }g\left(z,\tau \right)I\left(t-\tau \right)\;d\tau =g\left(z,t\right)*I\left(t\right)$$

The pressure response $p\left(z,t\right)$ is then received by a transducer and passes through a receiver-filter with an impulse response $r\left(t\right)$ (or, equivalently, transfer function $\tilde{R}\left(\omega \right)$).

## 3 Signal to noise ratio and resolution

**Fig. 2:**
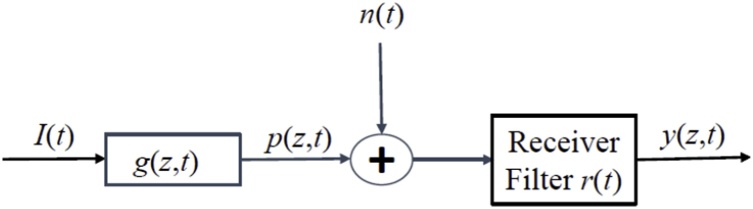
Photoacoustic measurement channel.

The photoacoustic measurement channel model is shown in Fig. 2. The finite energy signal $p\left(z,t\right)$ is received at the receiver in the presence of zero-mean Gaussian noise $n\left(t\right)$. The noise is assumed to be stationary and ergodic and to have a double-sided power spectral density of ${\tilde{S}}_{nn}\left(\omega \right)$. Furthermore, $n\left(t\right)$ is assumed to be statistically independent of both the transmitted input waveform $I\left(t\right)$ and absorber impulse response $g\left(z,t\right)$. Generally, the system impulse response $g\left(z,t\right)$ is not known a priori and the goal of the measurement process is to find it from the PA signal. The output of the receiver-filter is given by

(8) $$y\left(z,t\right)=y_{s}\left(z,t\right)+y_{n}\left(t\right)$$

where $y_{s}\left(z,t\right)$ is the pressure signal component and $y_{n}\left(t\right)$ is the noise signal component of the receiver output. These two components are given by

(9) $$\begin{array}{l} \;\;\;\;\;\;y_{s}\left(z,t\right)=r\left(t\right)*p\left(z,t\right)=r\left(t\right)*g\left(z,t\right)*I\left(t\right)\\ \text{or}\;\;\;{\tilde{y}}_{s}\left(z,\omega \right)=\tilde{R}\left(\omega \right)\tilde{G}\left(z,\omega \right)\tilde{I}\left(\omega \right) \end{array}$$

and

(10) $$\begin{array}{l} \;\;\;\;\;\;\;y_{n}\left(t\right)=r\left(t\right)*n\left(t\right)\\ \text{or}\;\;\;\;{\tilde{y}}_{n}\left(\omega \right)=\tilde{R}\left(\omega \right){\tilde{S}}_{nn}\left(\omega \right) \end{array}$$

When a waveform $y\left(z,t\right)$ is received, it may contain only noise or a pressure signal (resulting from the presence of a photoacoustic absorber) plus noise. For SNR optimization, given a chosen waveform/receiver filter pair, the instantaneous SNR at time $t_{0}$ for a measurement made at position *z* is defined as

(11) $$SNR={\left(\frac{S}{N}\right)}_{t_{0}}=\frac{{\left|y_{s}\left(z,t_{0}\right)\right|}^{2}}{E{\left|y_{n}\left(t_{0}\right)\right|}^{2}}$$

where $E\left|y_{n}\left(t_{0}\right)\right|$ refers to the noise expectation value.

The waveform design variables are then the waveform/receiver-filter pair $\left(I\left(t\right),r\left(t\right)\right)$ aimed to simultaneously achieve the desired SNR and resolution goals of the PA system.

Resolution is often introduced as the ability of a system to resolve point-like absorbers which are close together, and is sometimes defined in relation to the temporal width of the input pulse or the cut off frequency of the system. In order to quantify the PA system’s ability to resolve an extended absorber profile, the definition of resolution adopted in this paper will be taken as the error between the ideal impulse response and the obtained PA (after receiver-filter) system response. Further details are given in section 5.

A point-like target absorber located at $z_{0}$ is modeled as a Dirac-delta function in space, $A\left(z\right)=\delta \left(z-z_{0}\right)$. Its temporal response is referred to as the Point-Spread Function (PSF), so that Eq. (7) gives

(12) $$PSF\left(z,t\right)=\frac{p_{0}}{2c_{s}}\left\{\begin{array}{c} I\left(t-\frac{\left(z-z_{0}\right)}{c_{s}}\right)z>z_{0}\\ I\left(t+\frac{\left(z-z_{0}\right)}{c_{s}}\right)z<z_{0} \end{array}\right)$$

Eq. (12) implies that the (temporal) shape of the input pulse $I\left(t\right)$ is completely unchanged by a spatial point source – the resulting *PSF* pressure is a time-delayed version of $I\left(t\right)$. The full width at half maximum of the *PSF* is related to the ability of the PA system to resolve point-like absorbers which are close together.

### 3.1 SNR improvement via matched filtering

Given the photoacoustic measurement channel as shown in Fig. 2, an absorber impulse response $g\left(z,t\right)$, and white noise $n\left(t\right)$ with power density $N_{o}/2$, the maximum possible value of the SNR at time $t_{0}$ can be achieved by matched-filtering and is given by

(13) $$SNR\leq \frac{2}{N_{0}}E_{P}$$

where $E_{P}$ is the energy of the received pressure signal $\tilde{p}\left(z,\omega \right)=\tilde{G}\left(z,\omega \right)\tilde{I}\left(\omega \right)$. The receiver-filter transfer function $\tilde{R}\left(\omega \right)$ such that the SNR is maximized is given by

(14) $$\tilde{R}\left(\omega \right)=\alpha {\tilde{G}}^{*}\left(z,\omega \right){\tilde{I}}^{*}\left(\omega \right)e^{-i\omega t_{0}}$$

where α is a system related constant and starred superscripts denote complex conjugation. The proof of expressions (13) and (14) can be shown via an application of the Cauchy−Schwartz inequality and is demonstrated elsewhere [23]. However, Eq. (14) is not necessarily implementable for a typical PA system since $\tilde{G}\left(z,\omega \right)$, determined by the absorber profile, Eq.(5), is not known a priori.

Under the assumption of thermal and elastic confinement implied by Eq. (1), to achieve maximum SNR, it is necessary to maximize the energy of the pressure response. Faced with an unknown $\tilde{G}\left(z,\omega \right)$, an often-chosen alternative approach is to maximize the energy in the input waveform, $\tilde{I}\left(\omega \right)$. Increasing the peak power in $\tilde{I}\left(\omega \right)$ (with finite duration) leads to improvement in SNR, although the available or allowable peak power eventually puts a limit to this approach. Another way to increase signal energy is to increase the duration of the input signal, $I\left(t\right)$. However, increasing the duration of the signal leads to a loss of resolution [27]. This can be understood from the idea that to achieve good resolution, two closely placed source points must be separated, i.e. seen as two closely spaced points in the PA response, rather than one large object. The SNR/resolution dilemma is associated with the Rayleigh criterion which requires large optical energy for high SNR. This implies a long pulse in a CW laser system, thus decreasing the resolving power of the PA signal as per that criterion [27].

One approach often taken to address these conflicting requirements is to design the receiver-filter as a matched filter to the input (transmit) waveform [[18], [19], [20]], sometimes referred to as correlation processing because the output pressure is now cross-correlated with the input pulse. In this view, “matched filtering” means correlation with the input signal - the “matching” of the filter is made with the input signal rather than the output pressure signal (which is optimal). In the case of correlation processing, the receiver-filter is implemented as

(15) $$\tilde{R}\left(\omega \right)=\alpha {\tilde{I}}^{*}\left(\omega \right)\;e^{-i\omega t_{0}}$$

Under the condition of Eq.(15), Eq.(9) for the PA signal becomes

(16) $$\begin{array}{l} {\tilde{y}}_{s}\left(z,\omega \right)=\tilde{R}\left(\omega \right)\tilde{G}\left(z,\omega \right)\tilde{I}\left(\omega \right)\\ \;\;\;\;\;\;\;\;\;\;\;\;=\alpha \;\tilde{G}\left(z,\omega \right)\tilde{I}\left(\omega \right){\tilde{I}}^{*}\left(\omega \right)e^{-i\omega t_{0}} \end{array}$$

Eq. (16) can be interpreted in input/output form as a pressure response to an ‘input’ pulse ${\tilde{I}}^{SD}\left(\omega \right)$ where ${\tilde{I}}^{SD}\left(\omega \right)={\left|\tilde{I}\left(\omega \right)\right|}^{2}$ is the spectral energy density of $I\left(t\right)$. This perspective allows for a simple way of physically interpreting and analyzing the output of the receiver-filter as being the PA response signal to a synthesized ‘effective’ pulse ${\tilde{I}}^{SD}\left(\omega \right)$.

In this view of matched filtering, the *PSF* is now controlled by ${\tilde{I}}^{SD}\left(\omega \right)={\left|\tilde{I}\left(\omega \right)\right|}^{2}$. Then, the SNR is given by

(17) $$SNR=\frac{{\left|\int_{-\infty }^{\infty }\;\tilde{G}\left(z,\omega \right){\tilde{I}}^{SD}\left(\omega \right)\;d\omega \right|}^{2}}{N_{0}\pi \int_{-\infty }^{\infty }{\tilde{I}}^{SD}\left(\omega \right)d\omega }$$

If $\tilde{G}\left(z,\omega \right)$ is bandlimited so that the bulk of its energy is concentrated in the frequency band $\left[\omega_{0},\omega_{0}+W\right]$, it is clear from examining the numerator of Eq. (17), that to maximize SNR, $I\left(t\right)$ needs to be designed so that its power spectral density ${\tilde{I}}^{SD}\left(\omega \right)$ is concentrated in that same frequency band.

### 3.2 Special case: Pulse compression and chirp waveform optimization

The ability of the system to distinguish closely spaced point absorbers is controlled by the width of the point spread function, which is essentially the width of the input pulse. However, the SNR is controlled by the energy in the input pulse. Pulse compression provides a potential solution to the resolution/SNR dilemma. The linear chirp is one such waveform that can be compressed and is given by

(18) $$I_{T}\left(t\right)=\left\{\begin{array}{c} \cos\left(2\pi f_{0}t+\frac{\pi \Delta t^{2}}{T}\right)\;\;\;\;\;\left|t\right|<\frac{T}{2}\\ 0\;\;\;\;\;\left|t\right|>\frac{T}{2} \end{array}\right)$$

where *T* is the duration of the chirp. During the *T* second interval of the pulse, the instantaneous frequency changes linearly from $\left(f_{0}-\Delta /2\right)$ to $\left(f_{0}+\Delta /2\right)$. $f_{0}$ is referred to as the center frequency of the chirp. The bandwidth Δ (chirp sweep) is the difference between highest and lowest frequency in the frequency range. The chirp sweep rate is the rate of change of frequency, which for a linear chirp is a constant given by Δ/T. The chirp is determined by specifying the center frequency, $f_{0}$, duration T and (bandwidth) sweep, Δ. Any two of sweep, sweep rate and duration can be specified but in this paper we adopt the convention that sweep and duration are the controlling parameters. The spectral density ${\left|\tilde{I}\left(\omega \right)\right|}^{2}$ of the chirp can be roughly approximated as a rectangular function and is given by [28]

(19) $${\left|{\tilde{I}}_{T}\left(f\right)\right|}^{2}\approx \left\{\begin{array}{c} \frac{T}{4\Delta }\;\;\;\;\;\;\;f_{0}-\frac{\Delta }{2}\leq f\leq f_{0}+\frac{\Delta }{2}\\ 0\;\;\;\;\;\;\;\;\;\;\;\;\;\;\;\;\;\;\;\;\;\;\;\;\text{otherwise} \end{array}\right)$$

It is known from [29] that 98–99% of the chirp’s energy is confined to the frequency range given in Eq. (19) for time-bandwidth $\left(T\Delta \right)$ products that are larger than about 100. Almost 95% of the spectral energy is confined to the same frequency interval for time-bandwidth products as small as 10.

The total energy of chirp can be calculated from

(20) $$E_{I}=\int_{-\infty }^{\infty }{\left|{\tilde{I}}_{T}\left(f\right)\right|}^{2}df=\frac{1}{2\pi }\int_{-\pi \Delta }^{\pi \Delta }\frac{T}{4\Delta }d\omega =\frac{T}{4}$$

Eq. (20) implies that chirp duration is the only parameter that affects total energy delivered. However, a larger bandwidth implies a smaller spectral energy density. The corresponding time domain function to ${\left|\tilde{I}\left(\omega \right)\right|}^{2}$ can be calculated via auto-correlation. For the chirp given in Eq. (18), the autocorrelation has been shown to be well approximated by a sinc function with a main lobe of width 2/Δ [29], which can be taken as its effective duration. The compression ratio is defined as the ratio of the duration of the chirp (*T*) to its ‘effective’ (after auto correlation) duration 2/Δ, and is given by TΔ/2. Thus, the time-bandwidth product (or sometimes referred as “dispersion factor”) (TΔ) of the chirp determines the pulse compression ratio, which is the ratio of duration of original pulse (energy of the pulse) to the effective duration of the pulse (indirectly a measure of the potential resolution of the pulse). By properly choosing the chirp bandwidth, the effective pulse duration of the chirp can be controlled to a small value. Hence, the equivalent input pulse $I^{SD}\left(t\right)$ is compressed. In intuitive terms, the time-bandwidth product is thus a measure of the chirp’s ability to resolve the SNR/resolution dilemma since it measures the system’s ability to reduce pulse width. In principle the SNR and resolution of the PA signal can be both improved by using a matched-filter with a chirp waveform.

With an unknown $\tilde{G}\left(z,\omega \right)$, the matched-filter receiver transfer function is chosen to match the input waveform and the SNR with this matched-filter is given by Eq. (17). If linear frequency modulated chirp is chosen as the input waveform, then ${\left|\tilde{I}\left(\omega \right)\;\right|}^{2}$ can be roughly approximated by Eq. (19). Hence, it follows that the SNR for a linear frequency modulated chirp is approximately given by

(21) $$SNR=\frac{1}{\pi N_{0}}\frac{{\left|\int_{\omega_{0}-\Delta \pi }^{\omega_{0}+\Delta \pi }\frac{T}{4\Delta }\tilde{G}\left(z,\omega \right)\;d\omega \right|}^{2}}{\int_{\omega_{0}-\Delta \pi }^{\omega_{0}+\Delta \pi }\frac{T}{4\Delta }d\omega }=\frac{T}{4\pi N_{0}\Delta^{2}}{\left|\int_{\omega_{0}-\Delta \pi }^{\omega_{0}+\Delta \pi }\tilde{G}\left(z,\omega \right)\;d\omega \right|}^{2}$$

where $\omega_{0}+\Delta \pi$ and $\omega_{0}-\Delta \pi$ are the chirp sweep upper and lower cutoff frequencies in *rad/s.* Substituting Eq. (5), the 1D statement of the Fourier shell theorem, without the time delay factor (propagating wave), it follows that

(22) $$SNR=\frac{p_{0}^{2}T}{16\pi N_{0}c_{s}^{2}\Delta^{2}}\left\{\begin{array}{l} {\left|\int_{\omega_{0}-\Delta \pi }^{\omega_{0}+\Delta \pi }\hat{A}\left(-k\right)\;d\omega \right|}^{2}z>0\\ {\left|\int_{\omega_{0}-\Delta \pi }^{\omega_{0}+\Delta \pi }\hat{A}\left(k\right)\;d\omega \right|}^{2}z<0 \end{array}\right)$$

Eq. (22) clearly shows that (i) SNR is directly proportional to the chirp duration, as would be expected, because increasing chirp duration means increasing the total energy delivered; (ii) SNR is inversely proportional to the square of the chirp sweep, because increasing chirp sweep means distributing the total energy over a wider bandwidth; and (iii) SNR is directly proportional to the absorber spectrum that lies within the frequency interval bounded by the chirp sweep range $\left(f_{0}+\frac{\Delta }{2}\right)2\pi =\omega_{0}+\Delta \pi$ and $\left(f_{0}-\frac{\Delta }{2}\right)2\pi =\omega_{0}-\Delta \pi$, because putting chirp energy in frequency ranges where the absorber spectrum does not have any frequency content implies wasting the chirp energy in a frequency zone where the absorber cannot respond. Specific examples of absorbers are considered to enable a deeper physical comprehension of Eq. (22) in the following section.

## 4 Absorber spectral analysis of SNR

From Eq. (22), the effective spatial frequency spectrum of the absorber is a key part in determining the SNR. For example, for a square absorber that has a shape given by $A\left(z\right)=rect\left(\frac{z}{l}\right)\;$, where *l* is the thickness of the absorber, the spatial Fourier transform evaluated at the wave number $\hat{A}\left(k\right)$ is given by a *sinc* function which has a width of 2c/l (Hz) with most of its energy concentrated in the main lobe. Hence, the absorber can be roughly approximated as a bandlimited absorber with bandwidth $\Delta_{a}=2c/l$ (Hz) that would capture most of the energy (> 90%) in the main lobe of the *sinc*. A true bandlimited absorber is a square function in the spatial frequency domain. Although this kind of absorber does not exist in reality, it is helpful for analyzing the implications of bandlimitedness of the absorber on the SNR trend. For a quick analysis of implications on SNR, it is insightful to consider an absorber that can be considered as approximately bandlimited with a bandwidth $\Delta_{a}$ corresponding to a reasonable concentration of spectral energy (for example the main lobe in a sinc function).

According to Eq. (22), SNR is determined by the absorber frequency spectrum that lies within the frequency interval bounded by the chirp sweep range $f_{0}+\frac{\Delta }{2}$ and $f_{0}-\frac{\Delta }{2}$. Since integration implies the area under the curve, Eq. (22) can be rewritten as

(23) $$SNR=\frac{T\times C}{\Delta^{2}}\times {\left(OverlappingArea\right)}^{2}$$

where $C=\frac{p_{0}^{2}}{16\pi N_{0}c_{s}^{2}}$ is a constant factor and “overlapping area” denotes the absorber frequency spectrum that lies within the frequency interval bounded by the chirp sweep range.

**Fig. 3:**
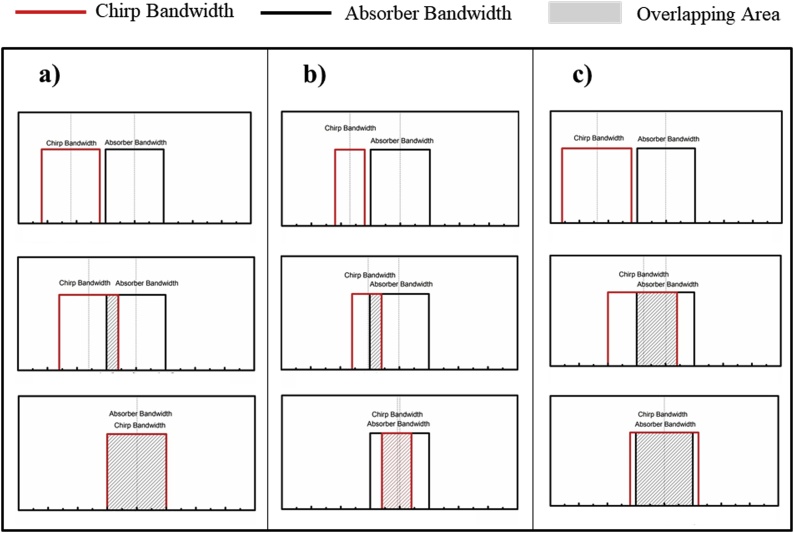
Overlapping conditions for bandlimited absorber for the cases where the chirp bandwidth a) equals, b) is smaller than, or c) is greater than, the absorber bandwidth.

For a bandlimited absorber, three different cases will be considered, $\Delta =\Delta_{a}$, $\Delta <\Delta_{a}$, and $\Delta >\Delta_{a}$. Fig. 3 demonstrates the effect of changing the center frequency of the chirp on the overlapping area (and hence SNR). The black square denotes the absorber frequency spectrum approximated as a bandlimited absorber, the red square denotes the chirp spectrum also approximated as a square in frequency, and the overlapping area in Eq. (23) is shown by the shaded area. The top row in Fig. 3 shows the no overlapping conditions which implies that the SNR is 0. The middle row in Fig. 3 shows the condition with partial overlapping, and the bottom row in Fig. 3 shows the ideal condition where the chirp spectrum lies either completely inside the absorber frequency spectrum or the absorber frequency spectrum lies completely inside the chirp spectrum.

It is obvious from Eq. (23) and Fig. 3 that as the chirp center frequency moves from the left of the absorber to the right of the absorber, the SNR will increase in the beginning until a maximum value is attained and then decrease until there is no overlapping area. The only difference between the three cases is the actual value of the maximum SNR. When $\Delta \leq \Delta_{a}$, the maximum SNR will be

(24) $$SNR_{MAX}=\frac{T\times C}{\Delta^{2}}{\left|\int_{-\frac{\Delta }{2}}^{\frac{\Delta }{2}}1df\right|}^{2}=T\times C$$

When $\Delta >\Delta_{a}$, the maximum SNR will be

(25) $$SNR_{MAX}=\frac{T\times C}{\Delta^{2}}{\left|\int_{-\frac{\Delta_{a}}{2}}^{\frac{\Delta_{a}}{2}}1df\right|}^{2}=T\times C\times \frac{\Delta_{a}^{2}}{\Delta^{2}}$$

It is noted that since $\Delta >\Delta_{a}$, the maximum SNR in Eq. (25), will be less than the value achieved in Eq. (24). A square absorber (square in space, sinc in frequency) can be considered to be approximately bandlimited. Therefore, its SNR trend should be similar to that of a bandlimited absorber. However, the overlapping area is slightly different from the bandlimited absorber.

In Fig. 3 a), the energy delivered by the chirp is constant from top to bottom, however the maximum SNR only occurs when the chirp completely overlaps the absorber spectrum, which means the incident energy is placed in the right frequency range. However, when the chirp frequency spectrum already covers most of the absorber frequency spectrum, increasing Δ effectively reduces the spectral energy density. From Fig. 3, it can be seen that increasing Δ does not increase the overlapping area, and due to the $\Delta^{2}$ in the denominator of Eq.(23), the SNR will start to decrease. More specifically, if comparing the bottom row in Fig. 3 a) and c), since the absorber frequency spectrum already completely overlaps the chirp spectrum as in Fig. 3 a), increasing the chirp bandwidth as in Fig. 3 c) will cause the SNR to decrease because chirp energy is wasted. In summary, to achieve optimal SNR, the key parameters of the chirp ($f_{0}$ and Δ) need to be controlled to place the chirp frequency spectrum (from $\left(f_{0}-\frac{\Delta }{2}\right)2\pi =\omega_{0}-\Delta \pi$ to $\left(f_{0}+\frac{\Delta }{2}\right)2\pi =\omega_{0}+\Delta \pi$) in the optimal frequency range where it can cover most of the absorber frequency spectrum.

## 5 Absorber profile and resolution

To quantify the quality of the cross-correlated signal at the output of the receiver-filter $y_{s}\left(t\right)$, an implementation of the concept of resolution is introduced. Since the goal of the pulse compression radar approach is to mimic a short effective pulse, for the purpose of the analysis herein we take as a measure of resolution the error between the signals obtained, $y_{s}\left(t\right)$ at the output of the receiver filter, and the ideal response $y_{ideal}\left(t\right)$, which is taken to be the impulse response to the system. The purpose of this definition of resolution is to examine the imaging ability of the PA system, rather than solely its ability to distinguish between closely spaced absorbers which is what would be implied if only the effective pulse width were taken as a measure of resolution. It will be shown in the analysis that follows that these two approaches to measuring resolution are not the same. In this paper, the photoacoustic system is simplified to the one-dimensional model of Fig. 1.

It is assumed that the media are acoustically homogeneous. The mathematical model for the absorbing medium can be expressed as a ‘square’ absorber. If light decay inside the absorber is taken as exponential (Beer-Lambert law), and assuming no scattering inside the absorber, then the absorber can be modeled as

(26) $$A\left(z\right)=rect\left(\frac{z}{l}\right)e^{-az}$$

where a is the optical attenuation coefficient.

In order to calculate the error between $y_{ideal}\left(t\right)$ and $y_{s}\left(t\right)$ after the receiver-filter, *N* points with uniform spacing are chosen between the two zero crossings of the impulse response curve. The error (maximum and average error) between the compressed pulse response and the ideal impulse response is used as a measure of the resolution of the pulse-compressed PAR system. The equation for calculating the maximum error is given by

(27) $${\left(Err\right|}_{\max}=\max\left|\frac{y_{s}\left(t_{i}\right)-y_{ideal}\left(t_{i}\right)}{y_{ideal}\left(t_{i}\right)}\right|\;\;\;\;\;\;i=1..N$$

where *N* is the number of sampling points. The equation for calculating the average error is given by

(28) $${\left(Err\right|}_{average}=\frac{1}{N}\sum_{i=1}^{N}\left|\frac{y_{s}\left(t_{i}\right)-y_{ideal}\left(t_{i}\right)}{y_{ideal}\left(t_{i}\right)}\right|$$

Eq.s (27) and (28) are used to calculate the errors (resolution) using simulations shown in the next section.

### 5.1 Cross-correlation signal of various absorber profiles

Prior to calculating the errors between the pulse-compressed result and the ideal impulse response, it is necessary to derive the closed form expression for the signal obtained after the receiver-filter, for the two signals corresponding to the aforementioned square and exponential decay absorbers. For a square absorber, the cross-correlated photoacoustic signal after the receiver-filter $y_{s}\left(z,t\right)$ is most easily expressed as

(29) $$y_{s}\left(z,t\right)=\frac{p_{0}}{2}\left\{\begin{array}{l} \int_{t+\frac{z}{c_{s}}-\frac{l}{2c_{s}}}^{t+\frac{z}{c_{s}}+\frac{l}{2c_{s}}}R_{II\_\cos}^{approx}\left(\tau \right)d\tau z<0\\ \int_{t-\frac{z}{c_{s}}-\frac{l}{2c_{s}}}^{t-\frac{z}{c_{s}}+\frac{l}{2c_{s}}}R_{II\_\cos}^{approx}\left(\tau \right)d\tau z>0 \end{array}\right)$$

Here, $R_{II\_\cos}^{approx}\left(t\right)$ is the (approximate) inverse Fourier transform of ${\left|\tilde{I}\left(\omega \right)\right|}^{2}$ where $\tilde{I}\left(\omega \right)$ is the Fourier transform of $I_{T}\left(t\right)$ given in Eq.(18) and has been shown to be well approximated by [29]

(30) $$R_{II\_\cos}^{approx}\left(t\right)=\left\{\begin{array}{c} T\frac{\sin\left(\pi \;\Delta \left|t\right|\right)}{\Delta \pi \left|t\right|}\cos\left(2\pi f_{0}t\right)\;\;\;\;\;\;\;\;-T\leq t\leq T\\ 0\;\;\;\;\;\;\;\;\;\;\;\;\;\;\;\;\;\;\;\;\;\;\;\;\;\;\;\;\;\;\;\;\;\;\text{otherwise} \end{array}\right)\;$$

The space variable z in Eq. (29) only depends on the measurement location (where the transducer is placed). Supposing that the measurement point is placed at $z<-\frac{l}{2}$ and is fixed, the signal $y_{s}\left(z,t\right)$ would be a function of t only, that is $y_{s}\left(t\right)$. The expression given by Eq. (29) for z<0 can be computed in closed form by the symbolic computer algebra system Maple (Maplesoft 2017). For the calculation in closed form, two different conditions are considered $\frac{l}{c_{s}}<\frac{T}{2}$ and $\frac{l}{c_{s}}>\frac{T}{2}$. The expression for $y_{s}\left(t\right)$ for $\frac{l}{c_{s}}<\frac{T}{2}$ is shown in Appendix A and for $\frac{l}{c_{s}}>\frac{T}{2}$ in Appendix B.

Similar to the approach used for the square wave absorber, the cross-correlated signal after the receiver-filter $y_{s}\left(z,t\right)$ can be computed for the exponential decay absorber described by Eq. (26) and is given by

(31) $$y_{s}\left(z,t\right)=\frac{p_{0}}{2}\left\{\begin{array}{l} \int_{t+\frac{z}{c_{s}}-\frac{l}{2c_{s}}}^{t+\frac{z}{c_{s}}+\frac{l}{2c_{s}}}R_{II\_\cos}^{approx}\left(\tau \right)e^{-ac_{s}\left(t-\tau \right)}d\tau z<0\\ \int_{t-\frac{z}{c_{s}}-\frac{l}{2c_{s}}}^{t-\frac{z}{c_{s}}+\frac{l}{2c_{s}}}R_{II\_\cos}^{approx}\left(\tau \right)e^{-ac_{s}\left(t-\tau \right)}d\tau z>0 \end{array}\right)$$

To obtain a closed-form analytic expression of Eq. (31) for z<0 via the symbolic computer algebra system Maple, in the exponential decay absorber case we need to separately consider the possibility of $f_{0}=\frac{\Delta }{2}$, as there is then a pole in the expression. Hence, another expression for the exponential decay absorber photoacoustic signal is required in the special case of $f_{0}=\frac{\Delta }{2}$. The expressions for $y_{s}\left(z,t\right)$ for the condition $\frac{l}{c_{s}}<\frac{T}{2}$ are given in Appendix C ($f_{0}\neq \frac{\Delta }{2}$) and Appendix D ($f_{0}=\frac{\Delta }{2}$). Similarly, for $\frac{l}{c_{s}}>\frac{T}{2}$, the response is given in Appendix E ($f_{0}\neq \frac{\Delta }{2}$) and Appendix F ($f_{0}=\frac{\Delta }{2}$).

### 5.2 Simulation results

In this section, several important parameters of the incident chirp will be investigated. This will provide a guide on how to choose the proper parameters of the chirp for the goal of obtaining a pressure response that will resemble the impulse response, or in other words, optimal resolution as defined in this paper. Furthermore, the SNR trends with different chirp parameters are also verified.

**Fig. 4:**
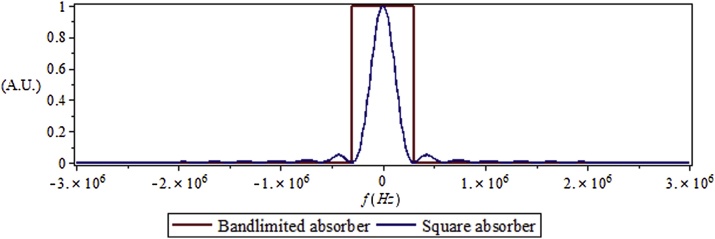
Absorber frequency spectrum.

Since the assumed expressions permit closed form results, the resolution of the system was measured as the error between the actual PA radar result and the desired impulse response (pulse PA response). The square and exponential decay absorbers are modeled with the parameter l=0.005(m). The transducer is assumed at position z=− 0.03(m). The exponential decay absorber is assumed with an absorption coefficient $a=200(m^{-1})$. The speed of sound in the scattering material and the absorber are assumed to be the same, and the value is taken as the speed of sound in water $c_{s}=1500(m/s)$. Fig. 4 shows the frequency spectrum of a square absorber with l=0.005(m) and $c_{s}=1500(m/s)$, which is the blue line. Most of the absorber energy is concentrated inside the frequency interval shown by the red line (Fig. 4). The absorber used in the simulations has most of its energy concentrated under 1 MHz, so the chirp parameters are chosen according to this order of magnitude. The initial pressure $p_{0}$, which merely serves as a scaling factor for simulations of the response, is chosen in Arbitrary Units (A.U.) as$p_{0}=10^{10}(A.U.)$.

#### 5.2.1 Effect of chirp duration t

As discussed earlier, the actual chirp duration is *T* and the compressed pulse duration is $\frac{2}{\Delta }$, which is referred to as the effective pulse duration. First, 4 sets of chirp parameters were tested with the chirp duration *T* chosen as $1\times 10^{-2}\;s$, $1\times 10^{-3}\;s$, $1\times 10^{-4}\;s$ and $1\times 10^{-5}\;s$. The other chirp parameters were held made constant at $\Delta =5.999\;MHz,\;f_{0}=3\;MHz$.

**Fig. 5:**
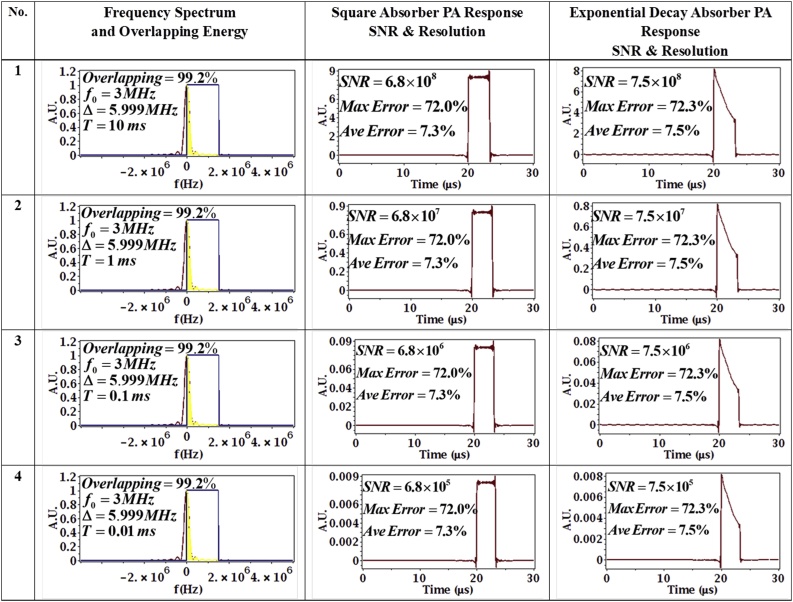
Effect of Chirp Duration on PA signal. The duration T is selected to be 10, 1, 0.1 and 0.01 ms (top to bottom), while the chirp bandwidth and center frequency are kept constant at $\Delta =5.999\;MHz,\;f_{0}=3\;MHz$. In column 2, the red line is the absorber spectrum, the blue line is the chirp spectrum (approximated as a square sweep for clarity) and the yellow denotes the overlap in the two spectra.

Since the chirp bandwidth Δ remains unchanged, the effective pulse duration $\frac{2}{\Delta }$ does not change, so it would be expected that there should not be any difference in resolution between the simulation results using these parameters, despite the changing duration of the chirp. However, the SNR is proportional to the chirp duration, *T*, so it would be expected that the SNR should decrease from parameter set 1–4 as *T* decreases because the total energy delivered decreases. Indeed, the simulation results for the PA signal after the receiver-filter showed no difference except for the amplitude between the 4 sets of parameters, as shown in Fig. 5. The overlapping area (the area filled with yellow in the second column of Fig. 5) of the frequency spectrum of the absorber and the chirp frequency spectrum plays the most important role in resolution. The absorber spectrum and chirp spectrum are both normalized to have unit amplitude. Since the chirp bandwidth and center frequency are constant through all 4 sets of parameters, implying that the overlapping area is constant (99.2% of the absorber energy lying inside the chirp spectrum), there is no doubt that the resolution error is also constant. The maximum error and average error were calculated using Eq. (27) and (28) with N=331 points, respectively, and have the same value for all 4 sets of parameters. The average errors were found to be more representative of the difference between PA signal and ideal impulse response since one sample point can lead to relatively large maximum errors despite the overall response generally being otherwise close to the impulse response. However, the maximum errors can reveal important problems in the PA signals so they were also calculated. The SNR was calculated through Eq. (22) and showed a linear relation with chirp duration *T*, as expected. The same SNR and resolution calculation approaches were used for all the simulations in this paper.

**Fig. 6:**
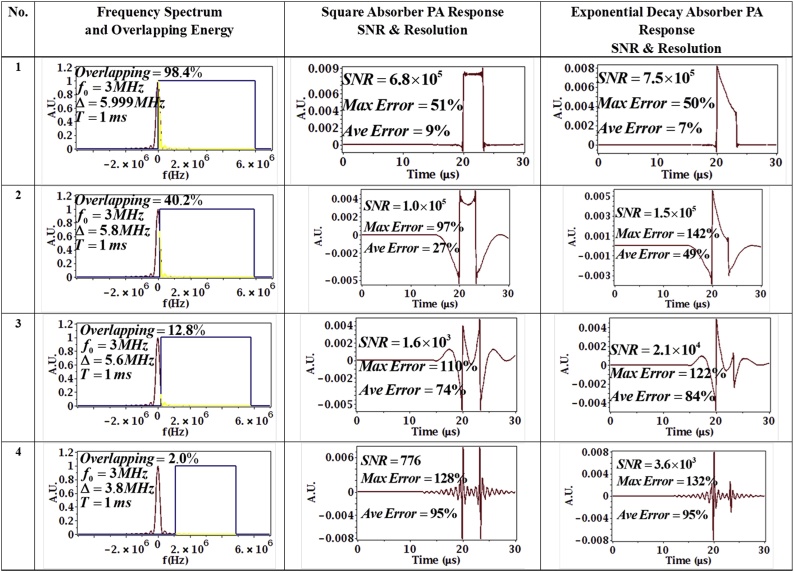
Effect of overlapping energy on absorber reconstruction error and SNR when the time-bandwidth product is kept constant. Center frequency is 3 MHz, parameters top to bottom are: T = 1 × 10^−5^swith Δ = 5.999 MHz; T = 1.034 × 10^−5^s with Δ = 5.8 MHz; T = 1.071 × 10^−5^s with Δ = 5.6 MHz, T = 1.579 × 10^−5^s with Δ = 3.8 MHz. In column 2, the red line is the absorber spectrum, the blue line is the chirp spectrum (approximated as a square sweep for clarity) and the yellow denotes the overlap in the two spectra.

For the second set of simulations, 4 sets of parameters were tested with a constant time-bandwidth product, with constant center frequency $f_{0}=3\;MHz$. In this case, as T increased, Δ decreased, but the time-bandwidth product (compression ratio) was held constant. Since the effective pulse duration (given by 2/Δ) was increased, this gave a PA signal that blurs the shape of the absorber, i.e. a worse resolution. The parameter sets are shown in Table 1. The simulation results are shown in Fig. 6 for both the square and exponential decay absorbers.

**Table 1:** Parameter Table with effective pulse duration changing.

Parameter sets No.	Chirp duration T (s)	Bandwidth Δ (MHz)
1	1 × 10^−5^	5.999
2	1.034 × 10^−5^	5.8
3	1.071 × 10^−5^	5.6
4	1.579 × 10^−5^	3.8

As can be seen from Fig. 6, the pressure response blurs the shape of the absorber when the bandwidth Δ decreases because the effective pulse duration $\frac{2}{\Delta }$ becomes large and can no longer be considered ‘short enough’ to represent a short pulse. The SNR of the PA signal also decreases with decreasing Δ because the chirp cannot overlap the energy concentration region of the absorber. As shown in Fig. 6, as the overlapping energy percentage becomes smaller, both the resolution and SNR decrease. Although the chirp duration *T* increased, which means more total energy delivered, the chirp cannot “catch” information about the absorber when it is sweeping the wrong range in the frequency domain, hence the resolution decreased. The correlation process in the receiver-filter also correlates the noise with the chirp waveform, so larger chirp energy also increases the noise level. Since the smaller bandwidth chirp only carries a small portion of useful signal energy, the SNR will decrease. The energy overlapping percentage is calculated for the square absorber and is also shown in Fig. 6. The exponential decay absorbers may have slightly different numbers, but the trend will be the same. For the square and exponential decay absorber, their frequency spectra are both centered at 0 *Hz*, but the chirp sweeps in the positive frequency region only. This follows because a negative frequency can be considered a phase change of the same positive frequency, hence covering the positive frequencies in the spectrum of an absorber is sufficient.

#### 5.2.2 Effect of chirp bandwidth

**Fig. 7:**
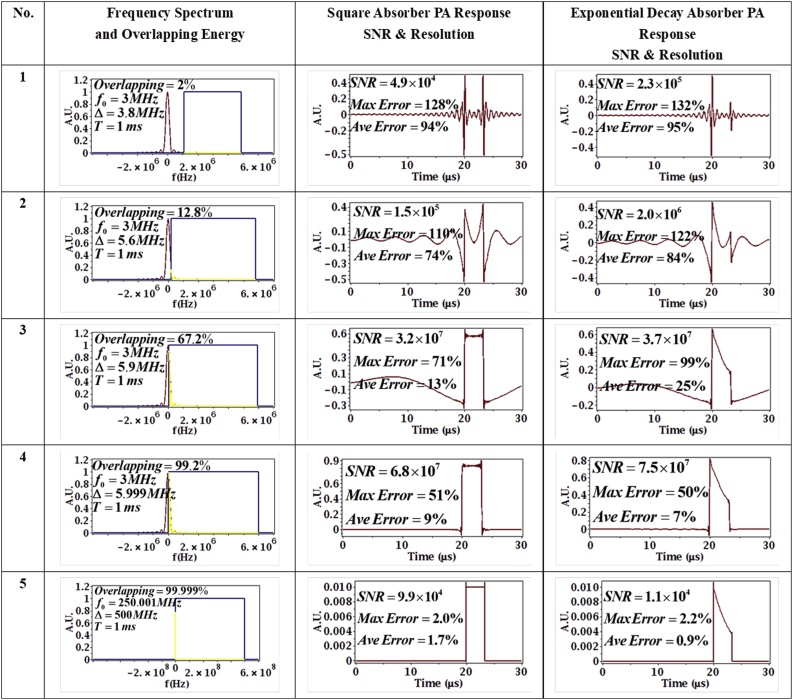
Effect of Bandwidth on PA signal. The bandwidth Δ is selected to be 3.8, 5.6, 5.9 5.999 and 500 MHz (top to bottom), while the chirp duration and center frequency are kept constant at $T=1\times 10^{-3}\;s,\;f_{0}=3\;MHz$ for the first 4 sets of parameters. The center frequency of the 5^th^ set of parameter is chosen to be 250.001 MHz to keep the frequency sweep range positive. In column 2, the red line is the absorber spectrum, the blue line is the chirp spectrum (approximated as a square sweep for clarity) and the yellow denotes the overlap in the two spectra.

In this subsection, the effects of the chirp frequency parameters on the PA signal are examined. The bandwidth Δ is the only parameter modified and is selected to be 3.8, 5.6, 5.9, 5.999 and 500 MHz, while the chirp duration and center frequency are kept constant at $T=1\times 10^{-3}\;s,\;f_{0}=3\;MHz$. The total energy of the chirp is constant with constant duration but the mean and peak power decrease with increased bandwidth. The simulation results are shown in Fig. 7 along with their associated normalized frequency overlapping conditions.

From Fig. 7, it can be observed that when Δ is large enough (or equivalently when the effective pulse duration is small enough), such as in the 4^th^ parameter set corresponding to 5.999 MHz, the PA signal resembles the absorber shape well. Hence, larger bandwidths are desirable in order to obtain narrower effective pulses and hence better resolutions. The large bandwidth of the pulsed laser approach gives good resolution because it is so wide (theoretically infinite) that it is guaranteed to “catch” information about the absorber everywhere as shown in the 5^th^ parameter set. However, many of those frequencies are likely wasted if (i) the absorber has no information to ‘provide’ in that frequency area (ii) if the transducer transfer function is not useful at some frequencies due to the inability of the transducer to respond. In the frequency domain, waveform engineering can tailor the frequency spectrum of the stimulus within the optimum response of the receiver transducers [20], as well as the optimum response of the absorber. We note that increasing the bandwidth Δ also results in a better SNR in the first 4 sets of simulation because the absorber frequency spectrum is centered at 0 *Hz* and the chirp center frequency is placed far from it. Hence, for this particular case, increasing Δ will cause the chirp (which is centered at $f_{0}=3MHz$ in the simulations) to cover more of the absorber frequency spectrum and hence a better SNR is obtained. However, when the chirp bandwidth is extremely large as shown by the 5^th^ parameter set, SNR will decrease because the chirp energy is wasted in the frequency range where the absorber does not have frequency content. Also, noise will increase as the wider spectral bandwidth of the incident pulse will contain more noise components.

#### 5.2.3 Effect of chirp center frequency

The other important parameter which affects the PA signal is the choice of chirp center frequency $f_{0}$. To analyze the effect of center frequency, the chirp duration and bandwidth were kept constant at $T=1\times 10^{-3}\;s$, Δ=3 MHz while only the chirp center frequency was set at 1.501, 1.55, 1.64, 1.8 and 2.5MHz. Hence, the effective pulse duration (given by $\frac{2}{\Delta }$) and time-bandwidth product (compression ratio) remained fixed. Hence, according to a traditional definition of resolution (the width of incident pulse [30]), we should expect to get similar results for these different parameters.

**Fig. 8:**
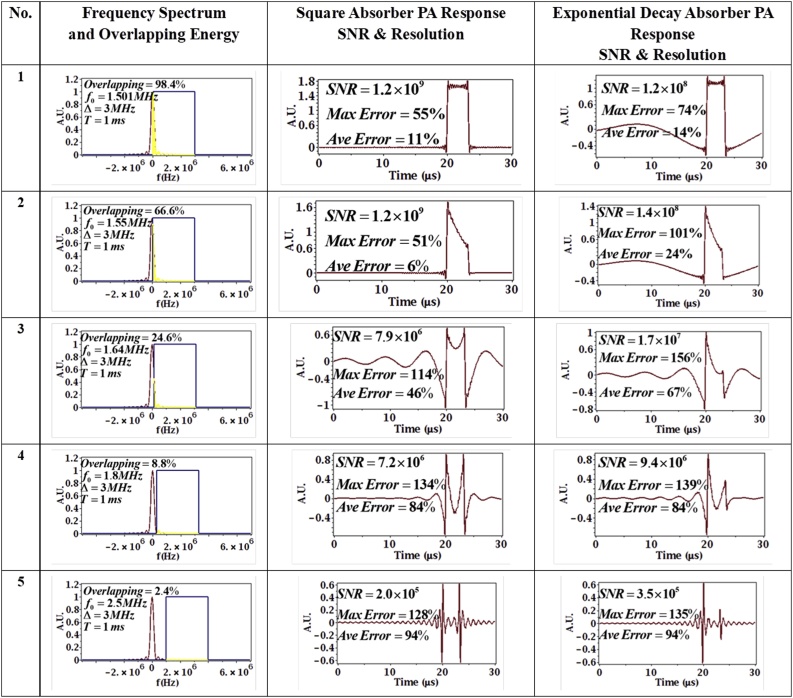
Effect of chirp center frequency on PA signal. Chirp duration and bandwidth are constant at $T=1\times 10^{-3}\;s$, Δ=3 MHz chirp center frequency is changed from 1.501, 1.55, 1.64, 1.8 and 2.5⁡MHz (top to bottom). In column 2, the red line is the absorber spectrum, the blue line is the chirp spectrum (approximated as a square sweep for clarity) and the yellow denotes the overlap in the two spectra.

In Fig. 8, from parameter sets 1–5, the chirp swept the same bandwidth (3 MHz) but in a different frequency range (moving away from the absorber center frequency). It is important to note that even if the chirp bandwidth (and thus the effective pulse duration which was 0.67 ms in this case) was the same in all the test cases, the results in Fig. 8 clearly show a large variability in resolution in the sense of the detailed evolution of the impulse response, with average errors changing from 6% in the best case to 94% in the worst case.

This addresses the comment made above about the lack of a standard definition of resolution. With a 3-MHz sweep and 0.67μs effective pulse width, from the resulting PA responses in Fig. 8, it is clear that the obtained responses vary from the expected impulse response to capturing only the edges of the absorber profile. Thus, the resolution (imaging ability) is clearly not the same in all the cases. As a result, it is suggested that an optimal resolution definition should involve the error to the desired impulse response/imaging ability of the system as shown in Eqs.(27) and (28). In particular, the average error, Eq.(28), between the desired impulse response and the obtained system response to a square absorber, emerges as a comprehensive measure of resolution. The square absorber in space is a good benchmark/reference absorber since the flat top of the absorber will test the system’s ability to resolve lower frequency (broad) details while the sharp corners of the square will test the system’s ability to resolve higher frequency details.

## 6 Summary and conclusions

In summary, this paper developed and analyzed a 1D theory of the PA radar, using frequency chirp modulation leading to pulse compression and input-pulse match filtering. Chirps make suitable waveform sources to achieve the goals of simultaneously improving SNR and optimal resolution with a moderate power and controllable frequency spectrum laser source, thus addressing some of the difficulties associated with pulsed lasers such as large bandwidth requirements and incident energy levels limited by safety standards. Closed form expressions for the compressed pulse and response to the compressed pulse were derived, which enabled a detailed analysis of the effects of the chirp parameters on both SNR and resolution. For the purpose of the analysis in this paper, resolution was defined as the error between the PA signal after the receiver-filter and an “ideal” pulse impulse response. The three key parameters to control the chirp are the duration *T*, center frequency $f_{0}$ and bandwidth Δ. Based on the analysis in this paper, several conclusions were reached: SNR is directly proportional to the duration of the chirp, inversely proportional to the square of the chirp sweep and directly proportional to the square of the ‘frequency overlap’ (or equivalently, energy coverage) of absorber and chirp. Increasing chirp sweep (bandwidth) increases SNR only as long as doing so implies increasing the ‘frequency overlap’ of the chirp and absorber. Once maximum frequency overlap has been achieved by the chirp sweep, increasing the sweep further only serves to reduce the SNR. Traditional measures of resolution in terms of duration or bandwidth of the input signal were found to not correlate with the imaging ability of the PA radar system. It was found that the average error between the ideal pulse impulse response and the obtained PAR system response to a square absorber can be used as a measure of spatial resolution.

When using correlation processing, the duration of the chirp has no effect on the resolution. The ‘effective duration’ of the post-correlation processed chirp is inversely proportional to the chirp sweep and is given by 2/Δ. However, an effective short duration of the chirp is insufficient to ensure a good resolution. The more of the frequency spectrum of the absorber the chirp frequency spectrum can cover, then better PA spatial resolution will ensue. The center frequency of the chirp determines the sweeping frequencies. When the chirp bandwidth is fixed, putting the center frequency at or close to the absorber bandwidth center frequency will give the best SNR, as this will maximize the overlapping area of spectral energy densities of absorber and chirp, as well as optimal resolution. The chirp center frequency $\left(f_{0}\right)$ and bandwidth $\left(\Delta \right)$ are the two parameters that most affect both SNR and resolution. Their proper selection can result in both good SNR and good absorber profile resolution. Optimal choices of both parameters are dependent on the spatial frequency spectrum of the absorber. However, in a realistic scenario (in vivo imaging), the absorber profile is always unknown. Difficulty in matching the chirp spectrum with absorber spectrum still exists. Future research on statistically determining the absorber profile for different kinds of carcinoma may help to address this problem.

## Transparency document

Transparency document

## Appendix A

The cross-correlated photoacoustic signal after the receiver-filter for square absorber under the condition $\frac{l}{c_{s}}<\frac{T}{2}$ is given by

(32) $$y_{s}\left(t\right)=\frac{p_{0}}{2}\left\{\begin{array}{l} 0t<-\frac{T}{2}\\ y_{s}^{1}\left(t\right)-\frac{T}{2}<t<\frac{l}{c_{s}}-\frac{T}{2}\\ y_{s}^{2}\left(t\right)\frac{l}{c_{s}}-\frac{T}{2}<t<0\\ y_{s}^{3}\left(t\right)0<t<\frac{l}{c_{s}}\\ y_{s}^{4}\left(t\right)\frac{l}{c_{s}}<t<\frac{T}{2}\\ y_{s}^{5}\left(t\right)\frac{T}{2}<t<\frac{l}{c_{s}}+\frac{T}{2}\\ 0\frac{T}{2}+\frac{l}{c_{s}}<t \end{array}\right)$$

The $y_{s}^{i}\left(t\right)$ in Eq. (32) are given by the following expressions.

(33) $$y_{s}^{1}\left(t\right)=\frac{p_{0}T}{2\Delta \pi }\left(Si\left(\frac{1}{2}T\Delta \pi -\pi Tf_{0}\right)+Si\left(\frac{1}{2}T\Delta \pi +\pi Tf_{0}\right)+Si\left(\Delta \pi t-2\pi f_{0}t\right)+Si\left(\Delta \pi t+2\pi f_{0}t\right)\right)$$

(34) $$\begin{array}{l} y_{s}^{2}\left(t\right)=\frac{p_{0}T}{2\Delta \pi }\left(Si\left(\Delta \pi t-2\pi f_{0}t\right)+Si\left(\Delta \pi t+2\pi f_{0}t\right)-Si\left(\Delta \pi t-2\pi f_{0}t-\Delta \pi \frac{l}{c_{s}}+2\pi f_{0}\frac{l}{c_{s}}\right)\right)\\ \left(-Si\left(\Delta \pi t+2\pi f_{0}t-\Delta \pi \frac{l}{c_{s}}-2\pi f_{0}\frac{l}{c_{s}}\right)\right) \end{array}$$

(35) $$\begin{array}{l} y_{s}^{3}\left(t\right)=\frac{p_{0}T}{2\Delta \pi }\left(Si\left(\Delta \pi t-2\pi f_{0}t\right)+Si\left(\Delta \pi t+2\pi f_{0}t\right)-Si\left(\Delta \pi t-2\pi f_{0}t-\Delta \pi \frac{l}{c_{s}}+2\pi f_{0}\frac{l}{c_{s}}\right)\right)\\ \left(-Si\left(\Delta \pi t+2\pi f_{0}t-\Delta \pi \frac{l}{c_{s}}-2\pi f_{0}\frac{l}{c_{s}}\right)\right) \end{array}$$

(36) $$\begin{array}{l} y_{s}^{4}\left(t\right)=\frac{p_{0}T}{2\Delta \pi }\left(Si\left(\Delta \pi t-2\pi f_{0}t\right)+Si\left(\Delta \pi t+2\pi f_{0}t\right)-Si\left(\Delta \pi t-2\pi f_{0}t-\Delta \pi \frac{l}{c_{s}}+2\pi f_{0}\frac{l}{c_{s}}\right)\right)\\ \left(-Si\left(\Delta \pi t+2\pi f_{0}t-\Delta \pi \frac{l}{c_{s}}-2\pi f_{0}\frac{l}{c_{s}}\right)\right) \end{array}$$

(37) $$\begin{array}{l} y_{s}^{5}\left(t\right)=\frac{p_{0}T}{2\Delta \pi }\left(Si\left(\frac{1}{2}T\Delta \pi -\pi Tf_{0}\right)+Si\left(\frac{1}{2}T\Delta \pi +\pi Tf_{0}\right)-Si\left(\Delta \pi t-2\pi f_{0}t-\Delta \pi \frac{l}{c_{s}}+2\pi f_{0}\frac{l}{c_{s}}\right)\right)\\ \left(-Si\left(\Delta \pi t+2\pi f_{0}t-\Delta \pi \frac{l}{c_{s}}-2\pi f_{0}\frac{l}{c_{s}}\right)\right) \end{array}$$

where Si(x) is the Sine Integral given by

(38) $$Si(x)=\int_{0}^{x}\frac{\sin\left(t\right)}{t}dt$$

## Appendix B

The cross-correlated photoacoustic signal after the receiver-filter for square absorber under the condition $\frac{l}{c_{s}}>\frac{T}{2}$ is given by

(39) $$y^{s}\left(t\right)=\frac{p_{0}}{2}\left\{\begin{array}{l} 0t<-\frac{T}{2}\\ y_{s}^{1}\left(t\right)-\frac{T}{2}<t<0\\ y_{s}^{2}\left(t\right)0<t<\frac{l}{c_{s}}-\frac{T}{2}\\ y_{s}^{3}\left(t\right)\frac{l}{c_{s}}-\frac{T}{2}<t<\frac{T}{2}\\ y_{s}^{4}\left(t\right)\frac{T}{2}<t<\frac{l}{c_{s}}\\ y_{s}^{5}\left(t\right)\frac{l}{c_{s}}<t<\frac{l}{c_{s}}+\frac{T}{2}\\ 0\frac{T}{2}+\frac{l}{c_{s}}<t \end{array}\right)$$

The $y_{s}^{i}\left(t\right)$ in Eq. (39) are given by the following expressions:

(40) $$y_{s}^{1}\left(t\right)=\frac{p_{0}T}{2\Delta \pi }\left(Si\left(\frac{1}{2}T\Delta \pi -\pi Tf_{0}\right)+Si\left(\frac{1}{2}T\Delta \pi +\pi Tf_{0}\right)+Si\left(\Delta \pi t-2\pi f_{0}t\right)+Si\left(\Delta \pi t+2\pi f_{0}t\right)\right)$$

(41) $$y_{s}^{2}\left(t\right)=\frac{p_{0}T}{2\Delta \pi }\left(Si\left(\frac{1}{2}T\Delta \pi -\pi Tf_{0}\right)+Si\left(\frac{1}{2}T\Delta \pi +\pi Tf_{0}\right)+Si\left(\Delta \pi t-2\pi f_{0}t\right)+Si\left(\Delta \pi t+2\pi f_{0}t\right)\right)$$

(42) $$\begin{array}{l} y_{s}^{3}\left(t\right)=\frac{p_{0}T}{2\Delta \pi }\left(Si\left(\Delta \pi t-2\pi f_{0}t\right)+Si\left(\Delta \pi t+2\pi f_{0}t\right)-Si\left(\Delta \pi t-2\pi f_{0}t-\Delta \pi \frac{l}{c_{s}}+2\pi f_{0}\frac{l}{c_{s}}\right)\right)\\ \left(-Si\left(\Delta \pi t+2\pi f_{0}t-\Delta \pi \frac{l}{c_{s}}-2\pi f_{0}\frac{l}{c_{s}}\right)\right) \end{array}$$

(43) $$\begin{array}{l} y_{s}^{4}\left(t\right)=\frac{p_{0}T}{2\Delta \pi }\left(Si\left(\frac{1}{2}T\Delta \pi -\pi Tf_{0}\right)+Si\left(\frac{1}{2}T\Delta \pi +\pi Tf_{0}\right)-Si\left(\Delta \pi t-2\pi f_{0}t-\Delta \pi \frac{l}{c_{s}}+2\pi f_{0}\frac{l}{c_{s}}\right)\right)\\ \left(-Si\left(\Delta \pi t+2\pi f_{0}t-\Delta \pi \frac{l}{c_{s}}-2\pi f_{0}\frac{l}{c_{s}}\right)\right) \end{array}$$

(44) $$\begin{array}{l} y_{s}^{5}\left(t\right)=\frac{p_{0}T}{2\Delta \pi }\left(Si\left(\frac{1}{2}T\Delta \pi -\pi Tf_{0}\right)+Si\left(\frac{1}{2}T\Delta \pi +\pi Tf_{0}\right)-Si\left(\Delta \pi t-2\pi f_{0}t-\Delta \pi \frac{l}{c_{s}}+2\pi f_{0}\frac{l}{c_{s}}\right)\right)\\ \left(-Si\left(\Delta \pi t+2\pi f_{0}t-\Delta \pi \frac{l}{c_{s}}-2\pi f_{0}\frac{l}{c_{s}}\right)\right) \end{array}$$

## Appendix C

The cross-correlated photoacoustic signal after the receiver-filter for exponential decay absorber under the condition $\frac{l}{c_{s}}<\frac{T}{2}$ and $f_{0}\neq \frac{\Delta }{2}$ is given by

(45) $$y_{s}\left(t\right)=\frac{p_{0}}{2}\left\{\begin{array}{l} 0t<-\frac{T}{2}\\ y_{s}^{1}\left(t\right)-\frac{T}{2}<t<\frac{l}{c_{s}}-\frac{T}{2}\\ y_{s}^{2}\left(t\right)\frac{l}{c_{s}}-\frac{T}{2}<t<0\\ y_{s}^{3}\left(t\right)0<t<\frac{l}{c_{s}}\\ y_{s}^{4}\left(t\right)\frac{l}{c_{s}}<t<\frac{T}{2}\\ y_{s}^{5}\left(t\right)\frac{T}{2}<t<\frac{l}{c_{s}}+\frac{T}{2}\\ 0\frac{T}{2}+\frac{l}{c_{s}}<t \end{array}\right)$$

The $y_{s}^{i}\left(t\right)$ in Eq. (45) are given by the following expressions:

(46) $$\begin{array}{l} y_{s}^{1}\left(t\right)=\frac{ip_{0}Te^{-ac_{s}t}}{4\Delta \pi }\left(-Ei\left(1,\frac{1}{2}ac_{s}T+\frac{1}{2}i\Delta \pi T+i\pi f_{0}T\right)+Ei\left(1,\frac{1}{2}ac_{s}T-\frac{1}{2}i\Delta \pi T+i\pi f_{0}T\right)\right)\\ -Ei\left(1,\frac{1}{2}ac_{s}T+\frac{1}{2}i\Delta \pi T-i\pi f_{0}T\right)+Ei\left(1,\frac{1}{2}ac_{s}T-\frac{1}{2}i\Delta \pi T-i\pi f_{0}T\right)\\ +Ei\left(1,-ac_{s}t-i\Delta \pi t-2i\pi f_{0}t\right)-Ei\left(1,-ac_{s}t+i\Delta \pi t-2i\pi f_{0}t\right)\\ \left(+Ei\left(1,-ac_{s}t-i\Delta \pi t+2i\pi f_{0}t\right)-Ei\left(1,-ac_{s}t+i\Delta \pi t+2i\pi f_{0}t\right)\right) \end{array}$$

(47) $$\begin{array}{l} y_{s}^{2}\left(t\right)=\frac{ip_{0}Te^{-ac_{s}t}}{4\Delta \pi }\left(-Ei\left(1,-i\Delta \pi t-2i\pi f_{0}t+i\Delta \pi \frac{l}{c_{s}}+2i\pi f_{0}\frac{l}{c_{s}}-ac_{s}t+al\right)\right)\\ +Ei\left(1,i\Delta \pi t-2i\pi f_{0}t-i\Delta \pi \frac{l}{c_{s}}+2i\pi f_{0}\frac{l}{c_{s}}-ac_{s}t+al\right)\\ -Ei\left(1,-i\Delta \pi t+2i\pi f_{0}t+i\Delta \pi \frac{l}{c_{s}}-2i\pi f_{0}\frac{l}{c_{s}}-ac_{s}t+al\right)\\ +Ei\left(1,i\Delta \pi t+2i\pi f_{0}t-i\Delta \pi \frac{l}{c_{s}}-2i\pi f_{0}\frac{l}{c_{s}}-ac_{s}t+al\right)\\ +Ei\left(1,-ac_{s}t-i\Delta \pi t-2i\pi f_{0}t\right)-Ei\left(1,-ac_{s}t+i\Delta \pi t-2i\pi f_{0}t\right)\\ \left(+Ei\left(1,-ac_{s}t-i\Delta \pi t+2i\pi f_{0}t\right)-Ei\left(1,-ac_{s}t+i\Delta \pi t+2i\pi f_{0}t\right)\right) \end{array}$$

(48) $$\begin{array}{l} y_{s}^{3}\left(t\right)=\frac{ip_{0}Te^{-ac_{s}t}}{4\Delta \pi }\left(-\ln\left(ac_{s}+i\Delta \pi +2i\pi f_{0}\right)+\ln\left(ac_{s}-i\Delta \pi +2i\pi f_{0}\right)\right)\\ -\ln\left(ac_{s}+i\Delta \pi -2i\pi f_{0}\right)+\ln\left(ac_{s}-i\Delta \pi -2i\pi f_{0}\right)\\ +\ln\left(-ac_{s}-i\Delta \pi -2i\pi f_{0}\right)-\ln\left(-ac_{s}+i\Delta \pi -2i\pi f_{0}\right)\\ +\ln\left(-ac_{s}-i\Delta \pi +2i\pi f_{0}\right)-\ln\left(-ac_{s}+i\Delta \pi +2i\pi f_{0}\right)\\ -Ei\left(1,-i\Delta \pi t-2i\pi f_{0}t+i\Delta \pi \frac{l}{c_{s}}+2i\pi f_{0}\frac{l}{c_{s}}-ac_{s}t+al\right)\\ +Ei\left(1,i\Delta \pi t-2i\pi f_{0}t-i\Delta \pi \frac{l}{c_{s}}+2i\pi f_{0}\frac{l}{c_{s}}-ac_{s}t+al\right)\\ -Ei\left(1,-i\Delta \pi t+2i\pi f_{0}t+i\Delta \pi \frac{l}{c_{s}}-2i\pi f_{0}\frac{l}{c_{s}}-ac_{s}t+al\right)\\ +Ei\left(1,i\Delta \pi t+2i\pi f_{0}t-i\Delta \pi \frac{l}{c_{s}}-2i\pi f_{0}\frac{l}{c_{s}}-ac_{s}t+al\right)\\ +Ei\left(1,-ac_{s}t-i\Delta \pi t-2i\pi f_{0}t\right)-Ei\left(1,-ac_{s}t+i\Delta \pi t-2i\pi f_{0}t\right)\\ \left(+Ei\left(1,-ac_{s}t-i\Delta \pi t+2i\pi f_{0}t\right)-Ei\left(1,-ac_{s}t+i\Delta \pi t+2i\pi f_{0}t\right)\right) \end{array}$$

(49) $$\begin{array}{l} y_{s}^{4}\left(t\right)=\frac{ip_{0}Te^{-ac_{s}t}}{4\Delta \pi }\left(-Ei\left(1,-i\Delta \pi t-2i\pi f_{0}t+i\Delta \pi \frac{l}{c_{s}}+2i\pi f_{0}\frac{l}{c_{s}}-ac_{s}t+al\right)\right)\\ +Ei\left(1,i\Delta \pi t-2i\pi f_{0}t-i\Delta \pi \frac{l}{c_{s}}+2i\pi f_{0}\frac{l}{c_{s}}-ac_{s}t+al\right)\\ -Ei\left(1,-i\Delta \pi t+2i\pi f_{0}t+i\Delta \pi \frac{l}{c_{s}}-2i\pi f_{0}\frac{l}{c_{s}}-ac_{s}t+al\right)\\ +Ei\left(1,i\Delta \pi t+2i\pi f_{0}t-i\Delta \pi \frac{l}{c_{s}}-2i\pi f_{0}\frac{l}{c_{s}}-ac_{s}t+al\right)\\ +Ei\left(1,-ac_{s}t-i\Delta \pi t-2i\pi f_{0}t\right)-Ei\left(1,-ac_{s}t+i\Delta \pi t-2i\pi f_{0}t\right)\\ \left(+Ei\left(1,-ac_{s}t-i\Delta \pi t+2i\pi f_{0}t\right)-Ei\left(1,-ac_{s}t+i\Delta \pi t+2i\pi f_{0}t\right)\right) \end{array}$$

(50) $$\begin{array}{l} y_{s}^{5}\left(t\right)=\frac{ip_{0}Te^{-ac_{s}t}}{4\Delta \pi }\left(-Ei\left(1,-i\Delta \pi t-2i\pi f_{0}t+i\Delta \pi \frac{l}{c_{s}}+2i\pi f_{0}\frac{l}{c_{s}}-ac_{s}t+al\right)\right)\\ +Ei\left(1,i\Delta \pi t-2i\pi f_{0}t-i\Delta \pi \frac{l}{c_{s}}+2i\pi f_{0}\frac{l}{c_{s}}-ac_{s}t+al\right)\\ -Ei\left(1,-i\Delta \pi t+2i\pi f_{0}t+i\Delta \pi \frac{l}{c_{s}}-2i\pi f_{0}\frac{l}{c_{s}}-ac_{s}t+al\right)\\ +Ei\left(1,i\Delta \pi t+2i\pi f_{0}t-i\Delta \pi \frac{l}{c_{s}}-2i\pi f_{0}\frac{l}{c_{s}}-ac_{s}t+al\right)\\ +Ei\left(1,-\frac{1}{2}ac_{s}T-\frac{1}{2}i\Delta \pi T-i\pi f_{0}T\right)-Ei\left(1,-\frac{1}{2}ac_{s}T+\frac{1}{2}i\Delta \pi T-i\pi f_{0}T\right)\\ \left(+Ei\left(1,-\frac{1}{2}ac_{s}T-\frac{1}{2}i\Delta \pi T+i\pi f_{0}T\right)-Ei\left(1,-\frac{1}{2}ac_{s}T+i\Delta \pi T+i\pi f_{0}T\right)\right) \end{array}$$

where $Ei\left(1,f\left(t\right)\right)=\int_{1}^{\infty }e^{-kf\left(t\right)}k^{-1}dk$ is the Exponential Integral.

## Appendix D

The cross-correlated photoacoustic signal after the receiver-filter for exponential decay absorber under the condition $\frac{l}{c_{s}}<\frac{T}{2}$ and $f_{0}=\frac{\Delta }{2}$ are still given by Eq. (45), where the $y_{s}^{i}\left(t\right)$ are given by the following expressions:

(51) $$\begin{array}{l} y_{s}^{1}\left(t\right)=\frac{ip_{0}Te^{-ac_{s}t}}{4\Delta \pi }\left(-Ei\left(1,\frac{1}{2}ac_{s}T+i\Delta \pi T\right)+Ei\left(1,\frac{1}{2}ac_{s}T-i\Delta \pi T\right)\right)\\ \left(+Ei\left(1,-ac_{s}t-2i\Delta \pi t\right)+Ei\left(1,-ac_{s}t+2i\Delta \pi t\right)\right) \end{array}$$

(52) $$\begin{array}{l} y_{s}^{2}\left(t\right)=\frac{ip_{0}Te^{-ac_{s}t}}{4\Delta \pi }\left(-Ei\left(1,-2i\Delta \pi t+2i\Delta \pi \frac{l}{c_{s}}-ac_{s}t+al\right)\right)\\ +Ei\left(1,2i\Delta \pi t-2i\Delta \pi \frac{l}{c_{s}}-ac_{s}t+al\right)\\ \left(+Ei\left(1,-ac_{s}t-2i\Delta \pi t\right)-Ei\left(1,-ac_{s}t+2i\Delta \pi t\right)\right) \end{array}$$

(53) $$\begin{array}{l} y_{s}^{3}\left(t\right)=\frac{ip_{0}Te^{-ac_{s}t}}{4\Delta \pi }\left(-\ln\left(ac_{s}+2i\Delta \pi \right)+\ln\left(ac_{s}-2i\Delta \pi \right)\right)\\ +\ln\left(-ac_{s}-2i\Delta \pi \right)-\ln\left(-ac_{s}+2i\Delta \pi \right)\\ -Ei\left(1,-2i\Delta \pi t+i\Delta \pi \frac{l}{c_{s}}-ac_{s}t+al\right)\\ +Ei\left(1,2i\Delta \pi t-2i\Delta \pi \frac{l}{c_{s}}-ac_{s}t+al\right)\\ \left(+Ei\left(1,-ac_{s}t-2i\Delta \pi t\right)-Ei\left(1,-ac_{s}t+2i\Delta \pi t\right)\right) \end{array}$$

(54) $$\begin{array}{l} y_{s}^{4}\left(t\right)=\frac{ip_{0}Te^{-ac_{s}t}}{4\Delta \pi }\left(-Ei\left(1,-2i\Delta \pi t+2i\Delta \pi \frac{l}{c_{s}}-ac_{s}t+al\right)\right)\\ +Ei\left(1,2i\Delta \pi t-2i\Delta \pi \frac{l}{c_{s}}-ac_{s}t+al\right)\\ \left(+Ei\left(1,-ac_{s}t-2i\Delta \pi t\right)-Ei\left(1,-ac_{s}t+2i\Delta \pi t\right)\right) \end{array}$$

(55) $$\begin{array}{l} y_{s}^{5}\left(t\right)=\frac{ip_{0}Te^{-ac_{s}t}}{4\Delta \pi }\left(-Ei\left(1,-2i\Delta \pi t+2i\Delta \pi \frac{l}{c_{s}}-ac_{s}t+al\right)\right)\\ +Ei\left(1,2i\Delta \pi t-2i\Delta \pi \frac{l}{c_{s}}-ac_{s}t+al\right)\\ \left(+Ei\left(1,-\frac{1}{2}ac_{s}T-i\Delta \pi T\right)-Ei\left(1,-\frac{1}{2}ac_{s}T+i\Delta \pi T\right)\right) \end{array}$$

## Appendix E

The cross-correlated photoacoustic signal after the receiver-filter for exponential decay absorber under the condition $\frac{l}{c_{s}}>\frac{T}{2}$ and $f_{0}\neq \frac{\Delta }{2}$ is given by

(56) $$y^{s}\left(t\right)=\frac{p_{0}}{2}\left\{\begin{array}{l} 0t<-\frac{T}{2}\\ y_{s}^{1}\left(t\right)-\frac{T}{2}<t<0\\ y_{s}^{2}\left(t\right)0<t<\frac{l}{c_{s}}-\frac{T}{2}\\ y_{s}^{3}\left(t\right)\frac{l}{c_{s}}-\frac{T}{2}<t<\frac{T}{2}\\ y_{s}^{4}\left(t\right)\frac{T}{2}<t<\frac{l}{c_{s}}\\ y_{s}^{5}\left(t\right)\frac{l}{c_{s}}<t<\frac{l}{c_{s}}+\frac{T}{2}\\ 0\frac{T}{2}+\frac{l}{c_{s}}<t \end{array}\right)$$

The $y_{s}^{i}\left(t\right)$ in Eq. (56) are given by the following expressions.

(57) $$\begin{array}{l} y_{s}^{1}\left(t\right)=\frac{ip_{0}Te^{-ac_{s}t}}{4\Delta \pi }\left(-Ei\left(1,\frac{1}{2}ac_{s}T+\frac{1}{2}i\Delta \pi T+i\pi f_{0}T\right)+Ei\left(1,\frac{1}{2}ac_{s}T-\frac{1}{2}i\Delta \pi T+i\pi f_{0}T\right)\right)\\ -Ei\left(1,\frac{1}{2}ac_{s}T+\frac{1}{2}i\Delta \pi T-i\pi f_{0}T\right)+Ei\left(1,\frac{1}{2}ac_{s}T-\frac{1}{2}i\Delta \pi T-i\pi f_{0}T\right)\\ +Ei\left(1,-ac_{s}t-i\Delta \pi t-2i\pi f_{0}t\right)-Ei\left(1,-ac_{s}t+i\Delta \pi t-2i\pi f_{0}t\right)\\ \left(+Ei\left(1,-ac_{s}t-i\Delta \pi t+2i\pi f_{0}t\right)-Ei\left(1,-ac_{s}t+i\Delta \pi t+2i\pi f_{0}t\right)\right) \end{array}$$

(58) $$\begin{array}{l} y_{s}^{2}\left(t\right)=\frac{ip_{0}Te^{-ac_{s}t}}{4\Delta \pi }\left(-\ln\left(ac_{s}+i\Delta \pi +2i\pi f_{0}\right)+\ln\left(ac_{s}-i\Delta \pi +2i\pi f_{0}\right)\right)\\ -\ln\left(ac_{s}+i\Delta \pi -2i\pi f_{0}\right)+\ln\left(ac_{s}-i\Delta \pi -2i\pi f_{0}\right)\\ +\ln\left(-ac_{s}-i\Delta \pi -2i\pi f_{0}\right)-\ln\left(-ac_{s}+i\Delta \pi -2i\pi f_{0}\right)\\ +\ln\left(-ac_{s}-i\Delta \pi +2i\pi f_{0}\right)-\ln\left(-ac_{s}+i\Delta \pi +2i\pi f_{0}\right)\\ -Ei\left(1,\frac{1}{2}ac_{s}T+\frac{1}{2}i\Delta \pi T+i\pi f_{0}T\right)+Ei\left(1,\frac{1}{2}ac_{s}T-\frac{1}{2}i\Delta \pi T+i\pi f_{0}T\right)\\ -Ei\left(1,\frac{1}{2}ac_{s}T+\frac{1}{2}i\Delta \pi T-i\pi f_{0}T\right)+Ei\left(1,\frac{1}{2}ac_{s}T-\frac{1}{2}i\Delta \pi T-i\pi f_{0}T\right)\\ +Ei\left(1,-ac_{s}t-i\Delta \pi t-2i\pi f_{0}t\right)-Ei\left(1,-ac_{s}t+i\Delta \pi t-2i\pi f_{0}t\right)\\ \left(+Ei\left(1,-ac_{s}t-i\Delta \pi t+2i\pi f_{0}t\right)-Ei\left(1,-ac_{s}t+i\Delta \pi t+2i\pi f_{0}t\right)\right) \end{array}$$

(59) $$\begin{array}{l} y_{s}^{3}\left(t\right)=\frac{ip_{0}Te^{-ac_{s}t}}{4\Delta \pi }\left(-\ln\left(ac_{s}+i\Delta \pi +2i\pi f_{0}\right)+\ln\left(ac_{s}-i\Delta \pi +2i\pi f_{0}\right)\right)\\ -\ln\left(ac_{s}+i\Delta \pi -2i\pi f_{0}\right)+\ln\left(ac_{s}-i\Delta \pi -2i\pi f_{0}\right)\\ +\ln\left(-ac_{s}-i\Delta \pi -2i\pi f_{0}\right)-\ln\left(-ac_{s}+i\Delta \pi -2i\pi f_{0}\right)\\ +\ln\left(-ac_{s}-i\Delta \pi +2i\pi f_{0}\right)-\ln\left(-ac_{s}+i\Delta \pi +2i\pi f_{0}\right)\\ -Ei\left(1,-i\Delta \pi t-2i\pi f_{0}t+i\Delta \pi \frac{l}{c_{s}}+2i\pi f_{0}\frac{l}{c_{s}}-ac_{s}t+al\right)\\ +Ei\left(1,i\Delta \pi t-2i\pi f_{0}t-i\Delta \pi \frac{l}{c_{s}}+2i\pi f_{0}\frac{l}{c_{s}}-ac_{s}t+al\right)\\ -Ei\left(1,-i\Delta \pi t+2i\pi f_{0}t+i\Delta \pi \frac{l}{c_{s}}-2i\pi f_{0}\frac{l}{c_{s}}-ac_{s}t+al\right)\\ +Ei\left(1,i\Delta \pi t+2i\pi f_{0}t-i\Delta \pi \frac{l}{c_{s}}-2i\pi f_{0}\frac{l}{c_{s}}-ac_{s}t+al\right)\\ +Ei\left(1,-ac_{s}t-i\Delta \pi t-2i\pi f_{0}t\right)-Ei\left(1,-ac_{s}t+i\Delta \pi t-2i\pi f_{0}t\right)\\ \left(+Ei\left(1,-ac_{s}t-i\Delta \pi t+2i\pi f_{0}t\right)-Ei\left(1,-ac_{s}t+i\Delta \pi t+2i\pi f_{0}t\right)\right) \end{array}$$

(60) $$\begin{array}{l} y_{s}^{4}\left(t\right)=\frac{ip_{0}Te^{-ac_{s}t}}{4\Delta \pi }\left(-\ln\left(ac_{s}+i\Delta \pi +2i\pi f_{0}\right)+\ln\left(ac_{s}-i\Delta \pi +2i\pi f_{0}\right)\right)\\ -\ln\left(ac_{s}+i\Delta \pi -2i\pi f_{0}\right)+\ln\left(ac_{s}-i\Delta \pi -2i\pi f_{0}\right)\\ +\ln\left(-ac_{s}-i\Delta \pi -2i\pi f_{0}\right)-\ln\left(-ac_{s}+i\Delta \pi -2i\pi f_{0}\right)\\ +\ln\left(-ac_{s}-i\Delta \pi +2i\pi f_{0}\right)-\ln\left(-ac_{s}+i\Delta \pi +2i\pi f_{0}\right)\\ -Ei\left(1,-i\Delta \pi t-2i\pi f_{0}t+i\Delta \pi \frac{l}{c_{s}}+2i\pi f_{0}\frac{l}{c_{s}}-ac_{s}t+al\right)\\ +Ei\left(1,i\Delta \pi t-2i\pi f_{0}t-i\Delta \pi \frac{l}{c_{s}}+2i\pi f_{0}\frac{l}{c_{s}}-ac_{s}t+al\right)\\ -Ei\left(1,-i\Delta \pi t+2i\pi f_{0}t+i\Delta \pi \frac{l}{c_{s}}-2i\pi f_{0}\frac{l}{c_{s}}-ac_{s}t+al\right)\\ +Ei\left(1,i\Delta \pi t+2i\pi f_{0}t-i\Delta \pi \frac{l}{c_{s}}-2i\pi f_{0}\frac{l}{c_{s}}-ac_{s}t+al\right)\\ +Ei\left(1,-\frac{1}{2}ac_{s}T-\frac{1}{2}i\Delta \pi T-i\pi f_{0}T\right)-Ei\left(1,-\frac{1}{2}ac_{s}T+\frac{1}{2}i\Delta \pi T-i\pi f_{0}T\right)\\ \left(+Ei\left(1,-\frac{1}{2}ac_{s}T-\frac{1}{2}i\Delta \pi T+i\pi f_{0}T\right)-Ei\left(1,-\frac{1}{2}ac_{s}T+i\Delta \pi T+i\pi f_{0}T\right)\right) \end{array}$$

(61) $$\begin{array}{l} y_{s}^{5}\left(t\right)=\frac{ip_{0}Te^{-ac_{s}t}}{4\Delta \pi }\left(-Ei\left(1,-i\Delta \pi t-2i\pi f_{0}t+i\Delta \pi \frac{l}{c_{s}}+2i\pi f_{0}\frac{l}{c_{s}}-ac_{s}t+al\right)\right)\\ +Ei\left(1,i\Delta \pi t-2i\pi f_{0}t-i\Delta \pi \frac{l}{c_{s}}+2i\pi f_{0}\frac{l}{c_{s}}-ac_{s}t+al\right)\\ -Ei\left(1,-i\Delta \pi t+2i\pi f_{0}t+i\Delta \pi \frac{l}{c_{s}}-2i\pi f_{0}\frac{l}{c_{s}}-ac_{s}t+al\right)\\ +Ei\left(1,i\Delta \pi t+2i\pi f_{0}t-i\Delta \pi \frac{l}{c_{s}}-2i\pi f_{0}\frac{l}{c_{s}}-ac_{s}t+al\right)\\ +Ei\left(1,-\frac{1}{2}ac_{s}T-\frac{1}{2}i\Delta \pi T-i\pi f_{0}T\right)-Ei\left(1,-\frac{1}{2}ac_{s}T+\frac{1}{2}i\Delta \pi T-i\pi f_{0}T\right)\\ \left(+Ei\left(1,-\frac{1}{2}ac_{s}T-\frac{1}{2}i\Delta \pi T+i\pi f_{0}T\right)-Ei\left(1,-\frac{1}{2}ac_{s}T+i\Delta \pi T+i\pi f_{0}T\right)\right) \end{array}$$

## Appendix F

The cross-correlated photoacoustic signal after the receiver-filter for exponential decay absorber under the condition $\frac{l}{c_{s}}>\frac{T}{2}$ and $f_{0}=\frac{\Delta }{2}$ is still given by Eq. (56) where the $y_{s}^{i}\left(t\right)$ are given by the following expressions.

(62) $$\begin{array}{l} y_{s}^{1}\left(t\right)=\frac{ip_{0}Te^{-ac_{s}t}}{4\Delta \pi }\left(-Ei\left(1,\frac{1}{2}ac_{s}T+i\Delta \pi T\right)+Ei\left(1,\frac{1}{2}ac_{s}T-i\Delta \pi T\right)\right)\\ \left(+Ei\left(1,-ac_{s}t-2i\Delta \pi t\right)-Ei\left(1,-ac_{s}t+2i\Delta \pi t\right)\right) \end{array}$$

(63) $$\begin{array}{l} y_{s}^{2}\left(t\right)=\frac{ip_{0}Te^{-ac_{s}t}}{4\Delta \pi }\left(-\ln\left(ac_{s}+2i\Delta \pi \right)+\ln\left(ac_{s}-2i\Delta \pi \right)\right)\\ +\ln\left(-ac_{s}-2i\Delta \pi \right)-\ln\left(-ac_{s}+2i\Delta \pi \right)\\ -Ei\left(1,i\Delta \pi t+\frac{1}{2}ac_{s}T\right)+Ei\left(1,-i\Delta \pi t+\frac{1}{2}ac_{s}t\right)\\ \left(+Ei\left(1,-ac_{s}t-2i\Delta \pi t\right)-Ei\left(1,-ac_{s}t+2i\Delta \pi t\right)\right) \end{array}$$

(64) $$\begin{array}{l} y_{s}^{3}\left(t\right)=\frac{ip_{0}Te^{-ac_{s}t}}{4\Delta \pi }\left(-\ln\left(ac_{s}+2i\Delta \pi \right)+\ln\left(ac_{s}-2i\Delta \pi \right)\right)\\ +\ln\left(-ac_{s}-2i\Delta \pi \right)-\ln\left(-ac_{s}+2i\Delta \pi \right)\\ -Ei\left(1,-2i\Delta \pi t+i\Delta \pi \frac{l}{c_{s}}-ac_{s}t+al\right)\\ +Ei\left(1,2i\Delta \pi t-2i\Delta \pi \frac{l}{c_{s}}-ac_{s}t+al\right)\\ \left(+Ei\left(1,-ac_{s}t-2i\Delta \pi t\right)-Ei\left(1,-ac_{s}t+2i\Delta \pi t\right)\right) \end{array}$$

(65) $$\begin{array}{l} y_{s}^{4}\left(t\right)=\frac{ip_{0}Te^{-ac_{s}t}}{4\Delta \pi }\left(-\ln\left(ac_{s}+2i\Delta \pi \right)+\ln\left(ac_{s}-2i\Delta \pi \right)\right)\\ +\ln\left(-ac_{s}-2i\Delta \pi \right)-\ln\left(-ac_{s}+2i\Delta \pi \right)\\ -Ei\left(1,-2i\Delta \pi t+2i\Delta \pi \frac{l}{c_{s}}-ac_{s}t+al\right)\\ +Ei\left(1,2i\Delta \pi t-2i\Delta \pi \frac{l}{c_{s}}-ac_{s}t+al\right)\\ \left(+Ei\left(1,-\frac{1}{2}ac_{s}t-i\Delta \pi T\right)-Ei\left(1,-\frac{1}{2}ac_{s}t+i\Delta \pi T\right)\right) \end{array}$$

(66) $$\begin{array}{l} y_{s}^{5}\left(t\right)=\frac{ip_{0}Te^{-ac_{s}t}}{4\Delta \pi }\left(-Ei\left(1,-2i\Delta \pi t+2i\Delta \pi \frac{l}{c_{s}}-ac_{s}t+al\right)\right)\\ +Ei\left(1,2i\Delta \pi t-2i\Delta \pi \frac{l}{c_{s}}-ac_{s}t+al\right)\\ \left(+Ei\left(1,-\frac{1}{2}ac_{s}T-i\Delta \pi T\right)-Ei\left(1,-\frac{1}{2}ac_{s}T+i\Delta \pi T\right)\right) \end{array}$$
