# Supplementary material for: Waveform engineering analysis of photoacoustic radar chirp parameters for spatial resolution and SNR optimization
Source: Photoacoustics. 2019 May 2;14:49–66. doi: 10.1016/j.pacs.2019.04.003 (PMC6517623; doi:10.1016/j.pacs.2019.04.003)
Supplement: Transparency document [file mmc1.zip › ConflictOfInterest_ZS.pdf]

Photoacoustics

Conflict of Interest Policy

Manuscript number (if applicable):<br>Article Title: Waveform Engineering Analysis of Photoacoustic Radar Chirp Parameters for Spatial Resolution and SNR Optimization	Author name: Zuwen Sun

Declarations

Photoacoustics requires that all authors sign a declaration of conflicting interests. If you have nothing to declare in any of these categories then this should be stated.

Conflict of Interest

A conflicting interest exists when professional judgement concerning a primary interest (such as patient's welfare or the validity of research) may be influenced by a secondary interest (such as financial gain or personal rivalry). It may arise for the authors when they have financial interest that may influence their interpretation of their results or those of others. Examples of potential conflicts of interest include employment, consultancies, stock ownership, honoraria, paid expert testimony, patent applications/registrations, and grants or other funding.

Please state any competing interests

The authors declare that there are no conflicts of interest.

Funding Source

All sources of funding should also be acknowledged and you should declare any involvement of study sponsors in the study design; collection, analysis and interpretation of data; the writing of the manuscript; the decision to submit the manuscript for publication. If the study sponsors had no such involvement, this should be stated.

Please state any sources of funding for your research

This research was financially supported by the Natural Sciences and Engineering Research Council of Canada, through Discovery grants to Natalie Baddour and Andreas Mandelis and through a CHRP Grant to Andreas Mandelis.

Signature (a scanned signature is acceptable, but each author must sign)

Print name: Zuwen Sun

孙祖文

Zuwen Sun
